# Evaluating the benefits and adverse effects of an enthracycline-taxane-capecitabine combined regimen in patients with early breast cancer

**DOI:** 10.18632/oncotarget.20386

**Published:** 2017-08-22

**Authors:** Jiantang Zhang, Fangmeng Fu, Yuxiang Lin, Yazhen Chen, Minjun Lu, Minyan Chen, Peidong Yang, Meng Huang, Chuan Wang

**Affiliations:** ^1^ Department of Breast Surgery, Affiliated Union Hospital of Fujian Medical University, Fuzhou, China; ^2^ Fujian Center for Disease Control and Prevention, China

**Keywords:** capecitabine, early breast cancer, disease-free survival, overall survival, side-effects

## Abstract

Capecitabine in addition to anthracycline-taxane based regimens for patients with early breast cancer (EBC) has been reported in previous clinical trials, but the reported efficacy of this regimen remained inconsistent. In order to clarify the survival benefit of this regimen, a meta-analysis was performed. The systematic literature search was conducted in PubMed, the Cochrane library and Google scholar. The hazard ratios (HRs) were used to evaluate the efficacy and adverse events. The result indicated that capecitabine combine with an anthracycline-taxane based regimen would significantly improve DFS (HR = 0.87, 95% CI 0.77–0.97) and OS (HR = 0.78, 95% CI 0.66–0.91) compared with the controls. In subgroup analysis, we found that capecitabine improved the DFS in hormone receptor negative (HR = 0.72, 95% CI 0.53–0.92) and triple negative (HR = 0.67, 95% CI 0.49–0.86) EBC patients. However, adding capecitabine might also increase the occurrence of some side-effects, such as hand-foot syndrome, stomatitis and diarrhea. Capecitabine combined with an anthracycline-taxane based regimen maybe effective and well-tolerated by patients with EBC, especially for triple negative breast cancer, and might be a good clinical choice.

## INTRODUCTION

Breast cancer (BC) is one of the most commonly diagnosed cancer among women in most countries, representing a quarter of all cancers diagnosed in women [1]. It is also the primary cause of cancer-related deaths among women [2]. Approximately three-quarters of BC patients are diagnosed at an early stage or are operable [3]. For these patients, it is essential to administer adjuvant chemotherapy to reduce the risk of recurrence [4–5]. Recent studies and meta-analyses have shown that the taxane-based (neo)adjuvant regimen reduce recurrence rates and improve survival in high-risk EBC patients [6–9].

Capecitabine is an oral prodrug of fluorouracil, it is metabolized in the liver and in cancerous tissue after absorption. Capecitabine is converted into 5-fluorouracil (5-FU) by sequential enzyme activity. The final process of capecitabine is converted to 5-FU through the enzymatic activity of thymidine phosphorylase (TP), which is known to be over expressed in tumor cells [10]. Capecitabine has been considered one of the most active drugs available for metastatic BC (MBC), and it has a favorable safety profile that is characterized by minimal alopecia and myelosuppression [11]. Previous phase III metastatic breast cancer trials had demonstrated that patients have a better survival benefit with capecitabine plus docetaxel versus docetaxel alone [12–13].

An athracycline-taxane based regimen is most frequently used for breast cancer (neo)adjuvant treatment. The efficacy and adverse effects of capecitabine in addition to this regimen in patients with EBC which were reported in previous trials remained inconsistent. The US Oncology Group trial revealed that the addition of capecitabine to an anthracycline-taxane regimen significantly improved the OS (HR = 0.68, 95% CI 0.51–0.92, *p* = 0.011) compared to the anthracycline-taxane regimen, but there was no significant difference in DFS (HR = 0.84, 95% CI 0.67–1.05, *p* = 0.125) between the two regimens [14]. At a 2016 American Society of Clinical Oncology (ASCO) conference, Shao Z. reported that the addition capecitabine to the adjuvant treatment of triple-negative patients could significantly improve RFS (HR = 0.57, 95% CI 0.33–1.0, *p* = 0.049). Whereas, no significant difference in DFS was found between adjuvant therapies with and without capecitabine (HR = 0.73, 95% CI 0.44–1.23, *p* = 0.2344) [15]. Contrary to these reports, other studies suggested that the addition of capecitabine to an anthracycline-taxane based regimen would not improve DFS and OS [16–19].

To determine whether EBC patients would benefit from capecitabine combined with an anthracycline-taxane based (neo)adjuvant regimen. We here report the results of a meta-analysis of seven prospective randomized controlled trials (RCTs), examining the efficacy and safety of (neo)adjuvant therapies with and without capecitabine for the treatment of early breast cancer patients.

## RESULTS

### Eligibility studies

**Figure 1:**
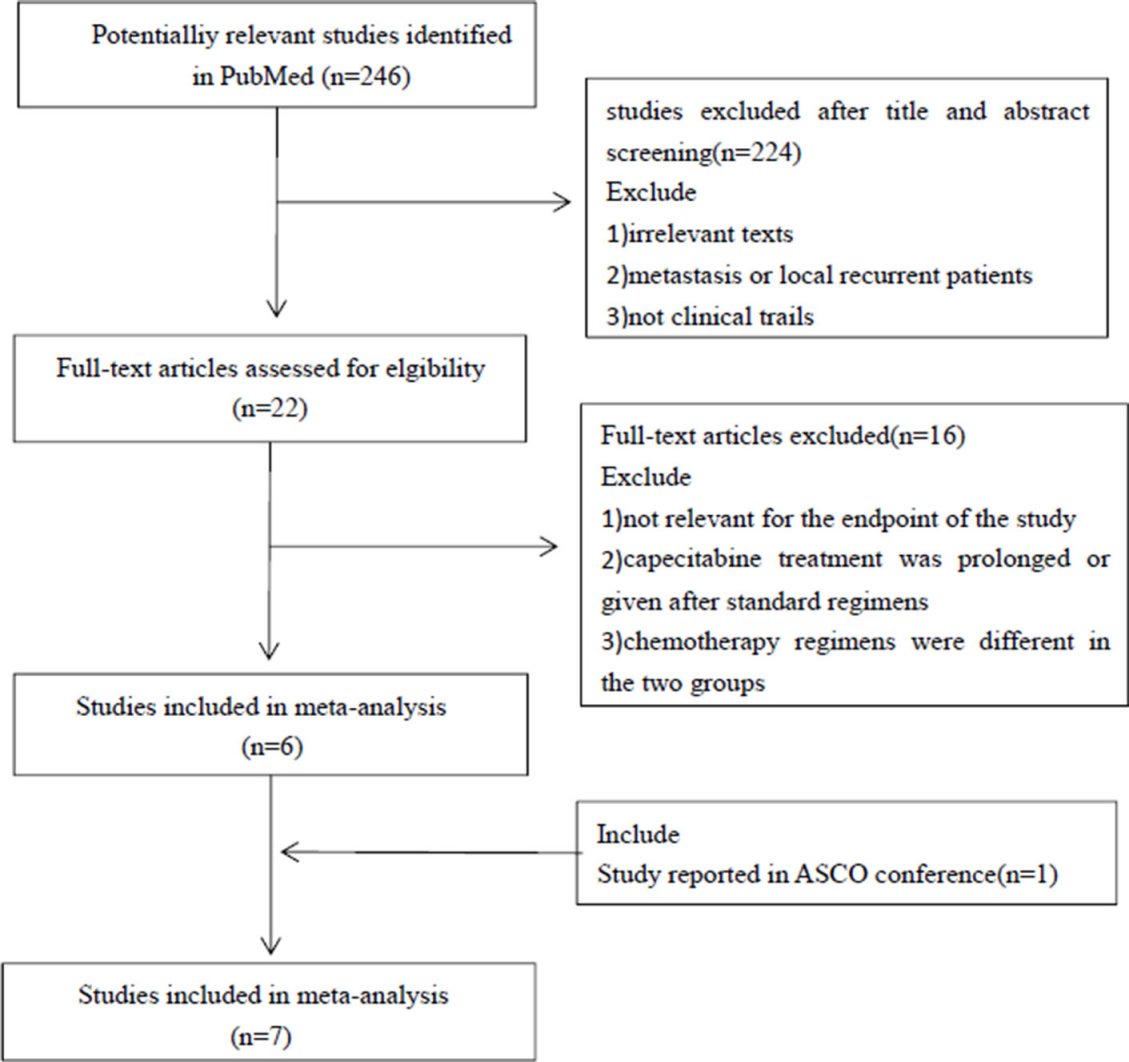
Process of studies selection

A total of seven trials (included ten articles) were identified from potential relevant studies (Figure 1). All the studies were randomized prospective cohort studies which included 7,416 early breast cancer patients; 3,725 received an anthracycline-taxane-capecitabine containing regimen and 3,691 received an anthracycline-taxane based regimen. Four trials focused on neoadjuvant chemotherapy and the other three were adjuvant chemotherapy trials. One trial was only published as an abstract and it only recruited triple negative early breast cancer patients. The remaining six trials were published as full articles. For the NSABP B-40 and GeparQuattro study, data were reported for three groups: Patients receiving gemcitabine in the NSABP B-40 trial and patients receiving docetaxel followed by capecitabine (T-X) in the GeparQuattro study were excluded from the meta-analysis based on the exclusion criteria. Of the seven trials, only six reported the survival data, four trials reported the endpoint pathologic complete response (pCR), and six trials reported adverse events. The characteristics of the seven RCTs included in this meta-analysis are shown in Table 1. The baseline demographics of patients of each eligible study are shown in Table 2.

**Table 1:** The characteristic of randomized clinical trials

Study	Aouthors	Year	Regimens	ITT	Dose of X	Dose of T	Follow-up
FinXX	Joensuu H	2012	TX-CEX	751	900 mg/m2 bid	60 mg/m2	5
			T-CEF	745		80 mg/m2	
US Oncology Group	O’Shaughnessy J	2015	AC-TX	1307	825 mg/m2 bid	75 mg/m2	5
			AC-T	1304		100 mg/m2	
Shao Z	Shao Z	2016	TX-CEX	288	1000 mg/m2 bid	N/A	2.5
			T-CEF	273		N/A	
Ohno S	Ohno S	2013	CEF-TX	239	825 mg/m2 bid	75 mg/m2	4.5
			CEF-T	238		75 mg/m2	
GeparQuattro	von Minckwitz G	2014	EC-TX	471	900 mg/m2 bid	75 mg/m2	5.4
			EC-T	471		100 mg/m2	
NSABP B-40	Bear HD	2015	TX-AC	400	825 mg/m2 bid	75 mg/m2	4.7
			T-AC	394		100 mg/m2	
ABCSG-24	Steger GG	2013	EDX	270	1000 mg/m2 bid	75 mg/m2	N/A
			ED	266		75 mg/m2	

ITT = intent-to-treat; X = capecitabine; T = docetaxel; C = cyclophosphamide; E = epeirubicin; A = doxorubicin; F = fluorouracil; D = docetaxel. N/A = Not available.

**Table 2:** Baseline demographics of patients of each eligible study

Study	Comparison	Mean Age, Years	HR positive, %	HR negative, %	HER-2 positive, %	HER-2 negative, %	TNBC, %
FinXX (2012)	TX-CEX vs T-CEF	52 vs 53	77 vs 75	23 vs 25	19 vs 19	81 vs 81	N/A
US Oncology Group (2015)	AC-TX vs AC-T	50 vs 51	64 vs 64	36 vs 36	12 vs 13	87 vs 86	30 vs 29
Shao Z (2016)	TX-CEX vs T-CEF	N/A	N/A	N/A	N/A	N/A	100 vs 100
Ohno S(2013)	CEF-TX vs CEF-T	49 vs 49	66 vs 66	32 vs 31	20 vs 19	75 vs 77	N/A
GeparQuattro (2014)	EC-TX vs EC-T	51 vs 49	64 vs 65	36 vs 35	31 vs 31	69 vs 69	23 vs 24
NSABP B-40 (2015)	TX-AC vs T-AC	N/A	59 vs 60	41 vs 40	0 vs 0	100 vs 100	N/A
ABCSG-24 (2013)	EDX vs ED	49 vs 49	67 vs 67	33 vs 33	74 vs 75	24 vs 23	N/A

X = capecitabine; T = docetaxel; C = cyclophosphamide; E = epeirubicin; A = doxorubicin; F = fluorouracil; D = docetaxel. N/A = Not available: HR = hormone receptor: TNBC = triple negative breast cancer.

### Total effect of capecitabine

**Figure 2:**
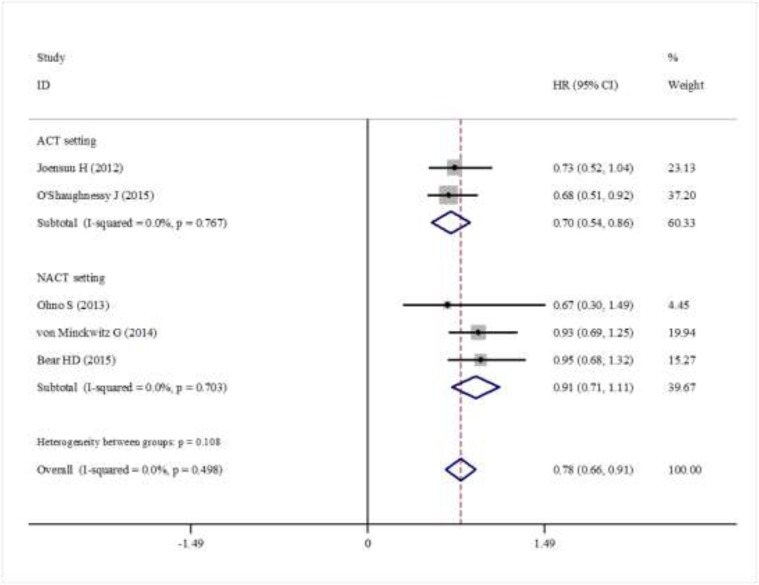
Forest plot showing the difference of total DFS in using capecitabine or not

**Figure 3:**
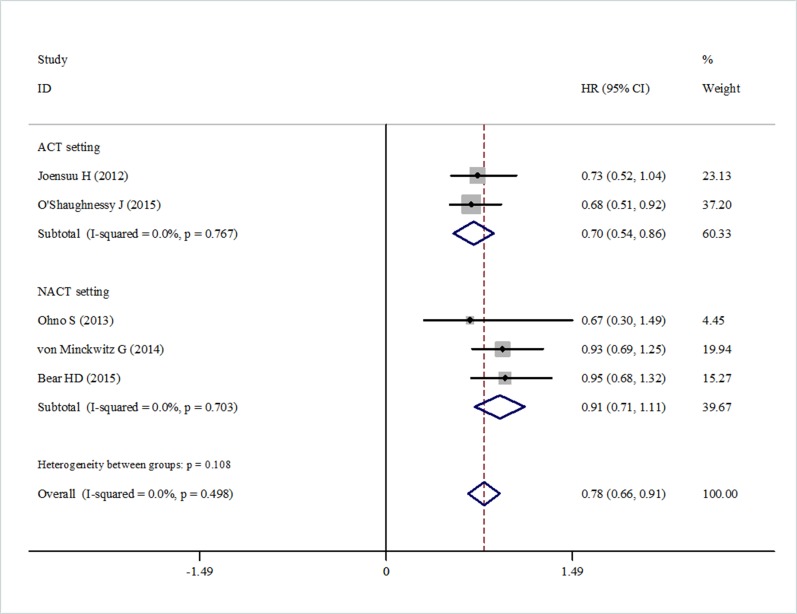
Forest plot showing the difference of total OS in using capecitabine or not

Data for DFS were reported in six trials. The pooled estimate suggested that capecitabine could significantly improve DFS (HR = 0.87, 95% CI 0.77–0.97, heterogeneity *I*^2^ = 0.0%, *p* = 0.833) compared with the controls (Figure 2). The OS data were reported in five trials. The pooled estimate showed that capecitabine could significantly improved OS (HR = 0.78, 95% CI 0.66–0.91, heterogeneity *I*^2^ = 0.0%, *p* = 0.449) compared with the no capecitabine arm (Figure 3).

**Figure 4:**
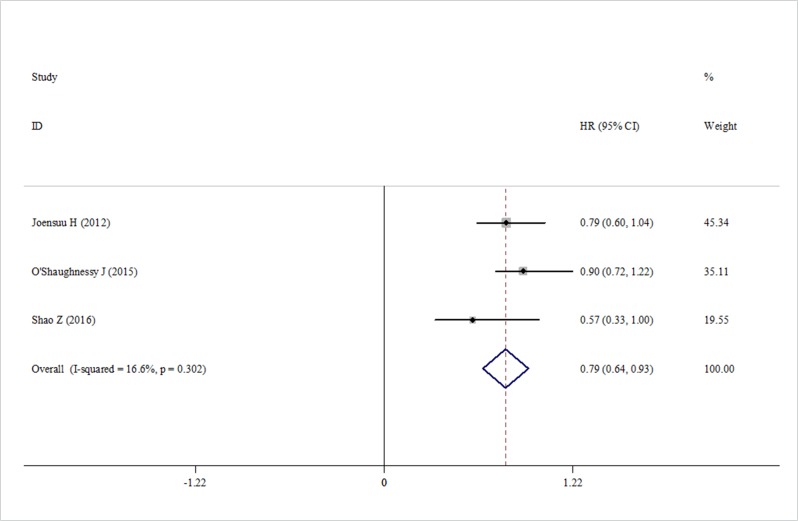
Forest plot of RFS in using capecitabine or not

**Figure 5:**
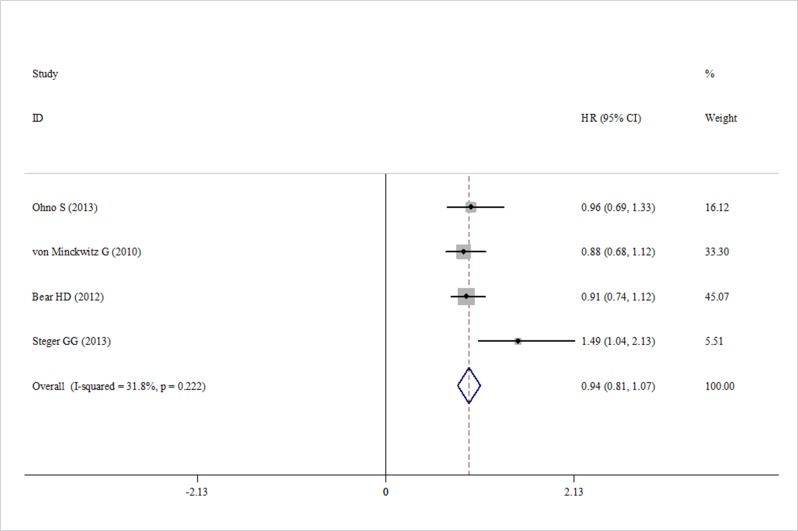
Forest plot of the pCR for the addition of capecitabine or not

Data about RFS were reported in three trials. We found that capecitabine significantly improved the RFS (HR = 0.79, 95% CI 0.64–0.93, heterogeneity *I*^2^ = 16.6%, *p* = 0.302) (Figure 4). Four trials reported the pCR rate [19, 20–22]. The pooled analysis showed no significant increase in the pCR rate in the capecitabine group (HR = 0.94, 95% CI 0.81–1.07, heterogeneity *I*^2^ = 31.8%, *p* = 0.222) compared with the control group (Figure 5).

### Subgroup analysis

**Figure 6:**
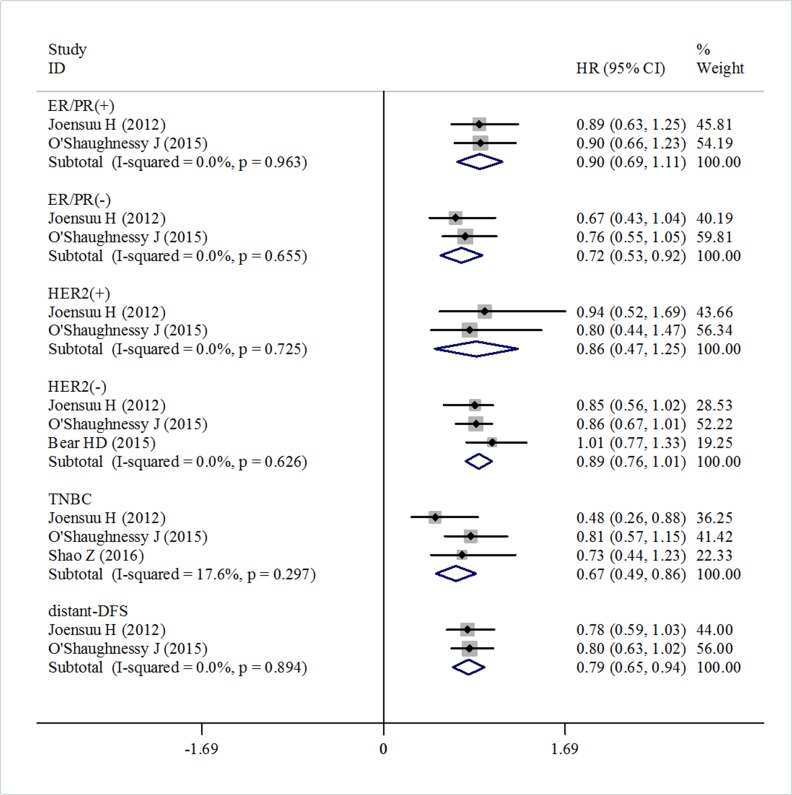
Forest plot of the subgroup DFS for the addition of capecitabine or not

Because of the lack of some patient information in trials, subgroup analysis was conducted only according to hormone receptor (HR) status, HER2 status, triple negative status, and for DFS, distant-DFS and breast cancer specific survival. The treatment effect according to HR status was only reported in two trials. The pooled estimate showed that there was no significant difference in the DFS in the HR positive subset between the capecitabine treated group and patients not treated with capecitabine (HR = 0.90, 95% CI 0.69–1.11, heterogeneity *I*^2^ = 0.0%, *p* = 0.963) (Figure 6). But for the HR negative subset, capecitabine significantly improved DFS (HR = 0.72, 95% CI 0.53–0.92, heterogeneity *I*^2^ = 0.0%, *p* = 0.655) (Figure 6).

The pooled data based on two trials suggested that there was no significant difference in DFS for HER2 positive patients who were treated with and without capecitabine (HR = 0.86, 95% CI 0.47–1.25, heterogeneity *I*^2^ = 0.0%, *p* = 0.725) (Figure 6). Similarly, the pooled analysis including three trials showed that capecitabine did not make a significant improvement in DFS in the HER2 negative subset compared with the control group (HR = 0.89, 95% CI 0.76–1.01, heterogeneity *I*^2^ = 0.0%, *p* = 0.626) (Figure 6).

Data for TNBC were reported in three trials. A pooled estimate suggested that the capecitabine group had significantly improved DFS (HR = 0.67, 95% CI 0.49–0.86, heterogeneity *I*^2^ = 17.6%, *p* = 0.297) compared with the no capecitabine group (Figure 6).

**Figure 7:**
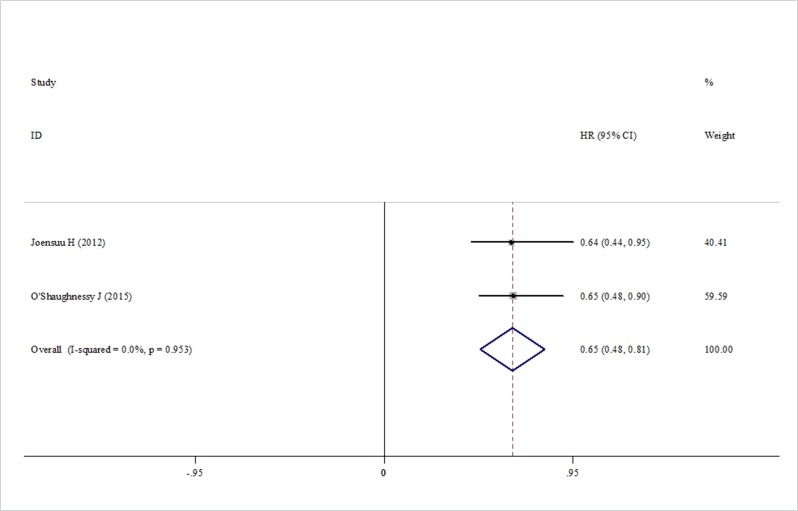
Forest plot of the breast cancer specific survival for the addition of capecitabine or not

Subgroup analysis based on two trials showed that adding capecitabine would significantly improve both distant-DFS and breast cancer specific survival compared with the controls. The pooled estimates were (HR = 0.79, 95% CI 0.65–0.94, heterogeneity *I*^2^ = 0.0%, *p* = 0.894) for distant-DFS (Figure 6) and (HR = 0.65, 95% CI 0.48–0.81, heterogeneity *I*^2^ = 0.0%, *p* = 0.953) for breast cancer specific survival (Figure 7), respectively.

### Safety

**Figure 8:**
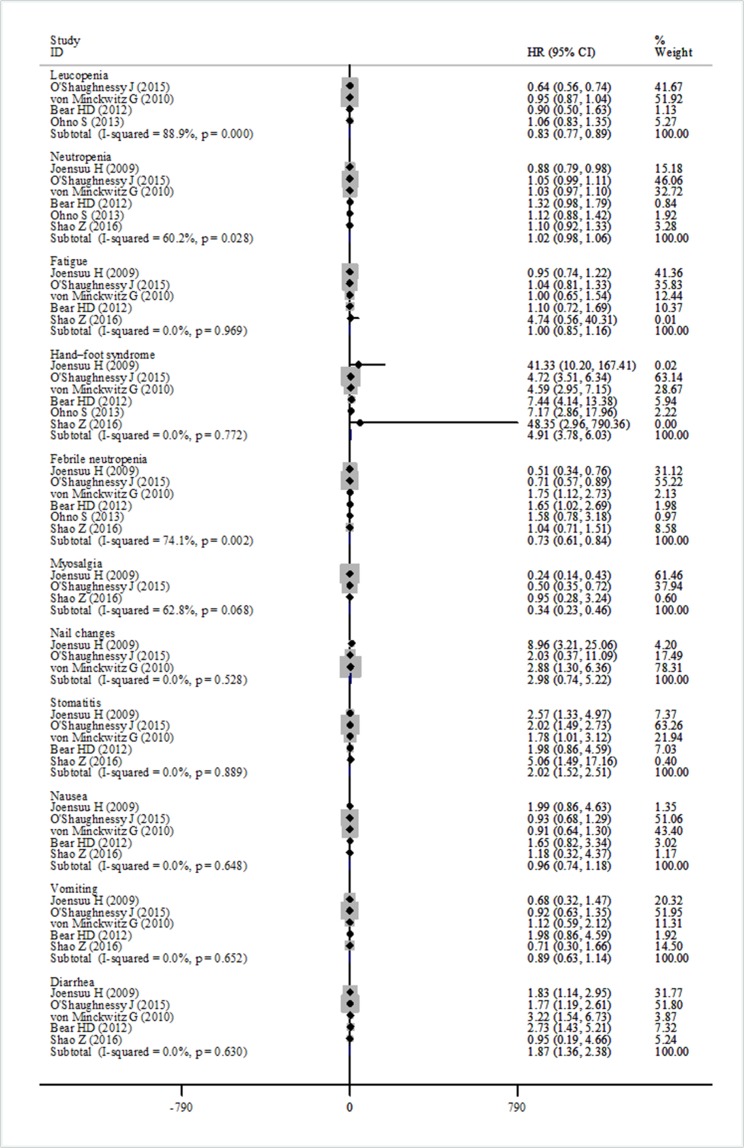
Forest plot of the adverse events for the addition of capecitabine or not

Adverse events were reported in six studies [14–15, 19, 21–23]. The pooled data showed a statistically significant increase in side effects in the capecitabine treated patients (≥ grade 3) among participants with early breast cancer: for hand-foot syndrome (HR = 4.91, 95% CI 3.78–6.03, heterogeneity *I*^2^ = 0.0%, *p* = 0.772), stomatitis (HR = 2.02, 95% CI 1.52–2.51, heterogeneity *I*^2^ = 0.0%, *p* = 0.889), diarrhea (HR = 1.87, 95% CI 1.36–2.38, heterogeneity *I*^2^ = 0.0%, *p* = 0.630). However, heterogeneity among trials was found in these analyses, possibly due to the use of different dosage of docetaxel and capecitabine (Figure 8).

### Publication bias

No publication bias was found between the included studies base on the funnel plots. (Supplementary Figure 1 and Supplementary Figure 2)

## DISCUSSION

A primary objective of this meta-analysis was to explore whether capecitabine in addition to an anthracycline-and taxane-based regimen can produce survival benefits in EBC patients. Our meta-analysis demonstrated that patients benefited substantially from capecitabine-anthracycline-taxane combined regimens in terms of DFS (HR = 0.87; 95% CI 0.77–0.97), RFS (HR = 0.79, 95% CI 0.64–0.93) and OS (HR = 0.78, 95% CI 0.66–0.91). In subgroup analysis, we found that capecitabine improved the DFS in hormone receptor negative (HR = 0.72, 95% CI 0.53–0.92) and triple negative (HR = 0.67, 95% CI 0.49–0.86) EBC patients.

Capecitabine is effective only after its conversion to 5-FU, but not by itself and its intermediates. Capecitabine is metabolized in the liver and in tumor tissue after absorption and is converted to 5-FU by sequential enzymatic activity. The final step is the conversion of 5′-deoxy-5-fluorouridine (5′-DFUR) to 5-FU through the enzymatic activity of thymidine phosphorylase (TP), an enzyme over expressed in tumor cells [10]. This tumor cells selective conversion of 5′-DFUR to 5-FU made greater efficacy and more safety than with other fluoropyrimidines. The capecitabine will have a synergistic effect with docetaxel and other cytotoxic drugs that can increase TP levels in the tumors, resulting from the direct chemical precursor of capecitabine being converted to 5-FU [24–25]. This synergistic effect should result in EBC patients benefiting from a capecitabine-taxane combined regimen.

Several studies have confirmed the efficacy of capecitabine treatment for patients with metastatic breast cancer [26–29]. After a median of 6.4 years followed-up, the US Oncology Group study have identified a significant improvement in OS (HR = 0.69, 95% CI 0.53–0.90) rather not in DFS (HR = 0.84, 95% CI 0.68–1.04) [14]. However, the data for 10-year survival from the randomized FinXX trial showed that capecitabine had no statistical significance during 10 years of follow-up in RFS (HR = 0.85 and 95% CI 0.68–1.07) or OS (HR = 0.83, 95% CI 0.65–1.06) [30]. This result was different from the conclusion of our meta-analysis, which may be related to the use of different doses of docetaxel in the control and experimental arms and approximately 24% of patients in the TX group that did not complete all cycles of treatment while about 47% of patients took less than the planned starting dose of capecitabine [14]. Data from a randomized controlled phase III study, GEICAM/2003-10, compared the ET-X (epirubicin and docetaxel followed by capecitabine) versus EC-T (epirubicin and cyclophosphamide followed by docetaxel) as adjuvant therapy for patients with node-positive EBC. The EC-T arm reported better invasive disease-free survival (IDFS) than the ET-X arm (HR = 1.30, 95% CI 1.03–1.64), but there was no significant difference in OS (HR = 1.13, 95% CI 0.81–1.55) [31]. However, this study used different chemotherapy regimens excepted capecitabine between the two arms, and cyclophosphamide was not used in the ET-X group. The authors considered that cyclophosphamide plays an important role in adjuvant BC therapy by its intrinsic antitumor properties or through the induction of amenorrhea in premenopausal patients [31]. All these factors may contribute to the opposite result in our study, and we excluded it in our meta-analysis base on the inclusion criteria. The recent CREAT-X trial also showed that capecitabine would significant improvement in DFS (HR = 0.70, 95% CI 0.53- 0.92) and OS (HR = 0.59, 95% CI 0.39- 0.90). However, capecitabine treatment was given after standard treatments (neoadjuvant chemotherapy and surgery) in the CREAT-X trial, and we excluded it in our meta-analysis base on the exclusion criteria [32].

Our data also showed that HR negative patients would have a DFS benefit with capecitabine over hormone receptor positive patients. Most patients in HR positive group accepted five years of treatment with tamoxifen or an aromatase inhibitor, which provides a significant survival benefits in patients with early-stage breast cancer [33] and reduces the risk of new contralateral breast tumors [34–35]. Moreover, different breast cancer subtypes have different recurrent times, the risk of HR positive breast cancer recurrence is for 8–9 years after surgery, but most included studies of this meta-analysis had failed to follow-up this long, and longer follow-up data are still warrented in future studies. While for HR negative patients, capecitabine would play a therapeutic role. The US Oncology Group study showed that capecitabine could significant improvement OS in HR negative patients (HR = 0.64, 95% CI 0.44–0.95) but not in the HR positive group [14]. The ABCSG24 trial also found that an addition of capecitabine to epirubicin and docetaxel had a greater chance of achieving pCR in the HR negative group [20]. But both the FinXX and US Oncology Group studies failed to indicate that capecitabine could improve DFS in HR negative group. CALGB 49907 trial showed that standard adjuvant chemotherapy with either CMF or doxorubicin plus cyclophosphamide is superior to capecitabine in older EBC patients, and it was pronounced in HR negative patients. These results just indicated that standard adjuvant chemotherapy is superior to capecitabine monotherapy in HR negative older patients with EBC. However, it may be more effective when capecitabine combined with standard adjuvant chemotherapy in HR negative older patients with EBC [36].

Triple negative breast cancer (TNBC) was defined by the absence of ER, PR and HER2. Patients with TNBC were more likely to have distant recurrence and death, and experienced higher risk of recurrence in the first 4 years after diagnosis compared to other breast cancer subtypes [37]. Thus, chemotherapy plays a crucial role for TNBC treatment, and it is the only systemic treatment for these patients. Our data showed that capecitabine combined with an anthracycline-taxane regimen would significantly improve DFS in TNBC patients. This result was similar with the FinXX trials, and after 10 years follow-up, the FinXX trials also showed more favorable RFS (HR = 0.43, 95% CI 0.24–0.79) and OS (HR = 0.55, 95% CI 0.31–0.96) in the capecitabine group [30]. The US Oncology Group study also found an OS benefit from capecitabine in TNBC patients (HR = 0.62, 95% CI 0.41–0.94) [14]. In the ABCSG-24 trial, the addition of capecitabine could significantly improve the pCR rate in TNBC patients (45.3% vs. 30.2%) [20]. The current study had showed that the expression of TP in TNBC was higher than other subtypes, indicating a better curative effect with capecitabine in patients with TNBC [38]. All these joining evidence demonstrated that TNBC patients would benefit from an anthracycline-taxane-capecitabine combined regimen. While Conversely, Shao Z. reported that the addition of capecitabine in combination with an anthracycline-taxane-based regimen for TNBC patients achieved no significant difference in the DFS between the groups (90.58% vs. 86.8%, *p* = 0.23), whereas that the RFS would be improved [18]. However, this study only included a 30 mouth follow-up and fewer events occurred. The short follow-up and fewer events may have substantially decreased the power of the study to show the superiority of the capecitabine arm. Our meta-analysis suggests that capecitabine added to anthracycline-taxane will be a appropriate regimen for adjuvant therapy of TNBC.

This meta-analysis suggested that capecitabine would not improve the pCR rate in the neoadjuvant chemotherapy setting(NACT), which was consistent with three of the included trials [17–19] while only the ABCSG study found that capecitabine could significantly improve the pCR rate in the neoadjuvant chemotherapy setting. This indicated that not all subtypes would benefit from capecitabine except for patients with TNBC, perhaps partly on account of a higher TP expression in TNBC [38]. Unfortunately, due to lack of subgroups data in most included trails, subgroups analysis were not available. Current trial-based meta-analyses indicated only a weak correlation between pCR and survival outcomes for patients with neoadjuvant treatment, while the prognostic value of pCR was demonstrated in some subtypes (triple negative, HER2-positive, and hormone receptor negative) [39–40]. And we also found that the DFS or OS were not statistically different between the two groups in the NACT setting. In contrast, several other neoadjuvant trials have reported improved survival outcomes in patients with pCR compared with those without pCR [41–42]. However, because of the lack of survival data in the ABCSG trial, our data only reveal a negative result. Therefore, more clinical trials are still needed to explore and improve the long term outcome data. Different from the other three included trials, [15–17], participants in the ABCSG study received the same dose of docetaxel in both the experimental and the control arms, and most patients (96% ED and 94% EDC) completed all six treatment cycles. These factors may have contributed to the significant benefit observed in the ABCSG trial [20]. Therefore, a appropriate dose of docetaxel and capecitabine is warrented in future studies, and the ABCSG trial might be considered as a reference.

Another result analysis shows that capecitabine would significantly improve both distant-DFS and breast cancer specific survival. A statistical significance was observed in two of these studies focusing on breast cancer specific survival and in one of these concerning about distant-DFS. Our data showed that capecitabine combined with an anthracycline-taxane based regimen would improve OS in EBC compared with the control group. This may due to capecitabine significantly improving distant-DFS and breast cancer specific survival, which indicated a lower rate of systemic recurrence [14].

Some toxicity was also shown in our data analysis. Adding capecitabine to a standard regimen would result in increases in some adverse events. The most frequently increased adverse events were hand-foot syndrome, stomatitis, and diarrhea. These results were consistent with the findings of the FinXX, US Oncology Group, GeparQuattro and NSABP B-40 trials. Although there was a higher incidence of grade 3/4 capecitabine-related adverse events, no new safety related events were found in the capecitabine group, and it has a favorable safety profile [14]. Unfortunately, a statistical heterogeneity was found in these analyses, which might be linked with the use of different dosages of capecitabine and docetaxel because of the toxicity observed in the individual studies. More clinical trials are still necessary to explore and improve the toxicity data.

This meta-analysis has some potential limitations. First, we did not include enough articles in our meta-analysis, because our study has strict execute and inclusion criteria, with one trial available only as a conference abstract. Second, the included trials were varied in their follow-up periods, therapy regimens, and dosages. Third, subgroup analyses were only reported in few trials, future clinical trials with adequate subgroup analyses are needed to report complete data.

Despite the limitations of our meta-analysis, the results strongly indicate that capecitabine combined with an anthracycline-taxane based (neo)adjuvant regimen will improve RFS, DFS and OS, and is well-tolerated by patients with early breast cancer. Moreover, the subgroups analyses also demonstrated that the capecitabine combined with an anthracycline-taxane based (neo)adjuvant regimen may be effective for some high risk early breast cancer subtypes, especially for triple negative breast cancer, and might be a proper clinical choice.

## MATERIALS AND METHODS

### Search strategy

The literature search was conducted in PubMed, Cochrane library and Google scholar. Conference abstracts were searched in American Society of Clinical Oncology (www.asco.org). The following search terms were used in PubMed and the Cochrane library; “capecitabine”, “adjuvant”, “neoadjuvant”, “chemotherapy” and the expanded MeSH term “breast neoplasms”. In Google scholar, we used the terms; “capecitabine”, “adjuvant”, “neoadjuvant”, “chemotherapy” and “breast cancer”. Relevant papers that were published before August 2016 were included. Moreover, the references of included literature and the related articles found in PubMed were also screened.

The criteria for inclusion studies were as follows: (a) randomized controlled prospective cohort trials comparing anthracycline-taxane based regimens with or without capecitabine; (b) early breast cancer patients without metastatic; (c) other chemotherapy regimens excepted capecitabine and the cycles of these drugs used were identical; (d) the primary endpoint was either disease-free survival (DFS), overall survival (OS) or pathologic complete response (pCR).

This systematic review excluded the relevant studies based on the following: (a) not a clinical trial; (b) metastasis or local recurrence breast cancer patients; (c) capecitabine treatment was prolonged or given after standard regimens; (d) not reporting the relevant endpoints of this systematic review.

### Outcome measures

The primary outcome of this meta-analysis was DFS, defined as the time between randomization and date of diagnosis of disease recurrent or metastatic spread, new primary cancer or any cause of death, whichever occurred first. The OS was defined as death from any cause at the study endpoint. The RFS was defined as the time from randomization to invasive breast cancer recurrence, or death from any cause. The pCR was defined as complete tumor regression with no invasive breast cancer cells in both primary tumors and regional lymph nodes. Other outcomes included distant-DFS (time to systemic metastases and deaths), breast cancer specific survival (time to death due to breast cancer).

### Data extraction

Data extraction from each eligible trial was used data extraction form. The following data was extracted: general characteristics of the study (the name of first author, the journal and year of publication, country, study design, the median follow-up time, the number of patients in each group), chemotherapeutic regimens and dose, HRs and 95% CIs for study outcomes, the number of patients with the grade 3 to 4 drug-related toxicities or pCR (the number of patients in each group was directly extracted from the included clinical trials or calculated according to the percentages).

### Quality assessment

The Jadad Quality Assessment Scale for cohort studies was used to assess the quality of the inclusive trials in our analysis, and including the follow items: methods of randomization, allocation concealment, blinding status and results (withdrawals and dropouts) [43] (Table 3).

**Table 3:** The quality evaluation of included clinical trials by Jadad scale

Study	randomization	Allocation concealment	blindness	result	score
FinXX	2	1	0	1	4
USON	1	1	0	1	3
Shao Z	2	1	0	0	3
Ohno S	1	1	0	1	3
GeparQuattro	1	1	0	1	3
NSABP B-40	2	1	0	1	4
ABCSG-24	2	1	0	1	4

### Statistical analysis

The primary efficacy outcome of our meta-analysis was DFS. And the secondary endpoints were OS, RFS, pCR. Outcomes measures were based on the intention-to-treat (ITT) analysis. Efficacy and adverse events analysis was compared between the two groups used the pooled log hazard ratios (HRs), and their 95% confidence intervals (CI) were also provided. The subgroup analyses were performed by including the HR status, HER2 status, triple negative status, distant-DFS, and breast cancer specific survival. A random effects model was used for drawing forest plots which are designed to demonstrate data distribution of the included articles. It was considered statistically significant if the 95% CI did not include the value 1. The between-study heterogeneity was tested with *I*-squared statistic and *p* values. When the *I*-squared was greater than or equal to 50%, or the *p* value was less than 0.05, we considered the presence of a high level of heterogeneity.

The probability of publication bias was assessed with the funnel plots and the Egger's test with statistical significance set at *p* < 0.05. All statistical analyses were performed using Stata (version 12.0).

## SUPPLEMENTARY MATERIALS FIGURES AND TABLES


