# Supplementary material for: Evaluating the benefits and adverse effects of an enthracycline-taxane-capecitabine combined regimen in patients with early breast cancer
Source: Oncotarget. 2017 Aug 22;8(46):81636–48. doi: 10.18632/oncotarget.20386 (PMC5655315; doi:10.18632/oncotarget.20386)
Supplement: Supplementary file 1 [file oncotarget-08-81636-s001.pdf]

Evaluating the benefits and adverse effects of an enthracycline-taxane-capecitabine combined regimen in patients with early breast cancer

SUPPLEMENTARY MATERIALS

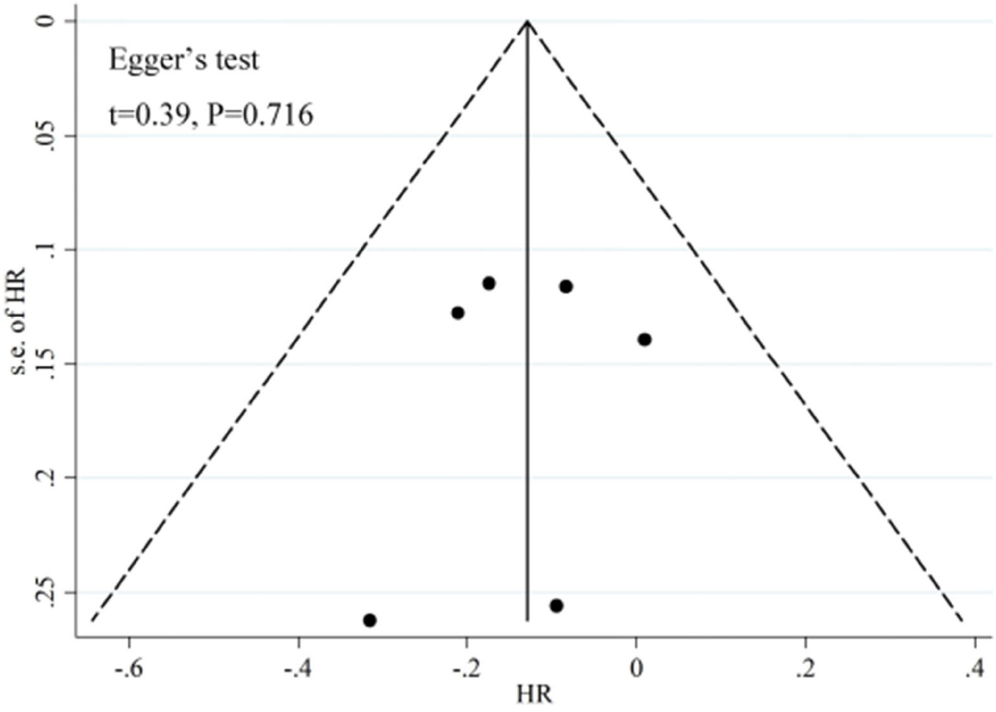

Supplementary Figure 1: Funnel plot of publication bias in DFS for the included.

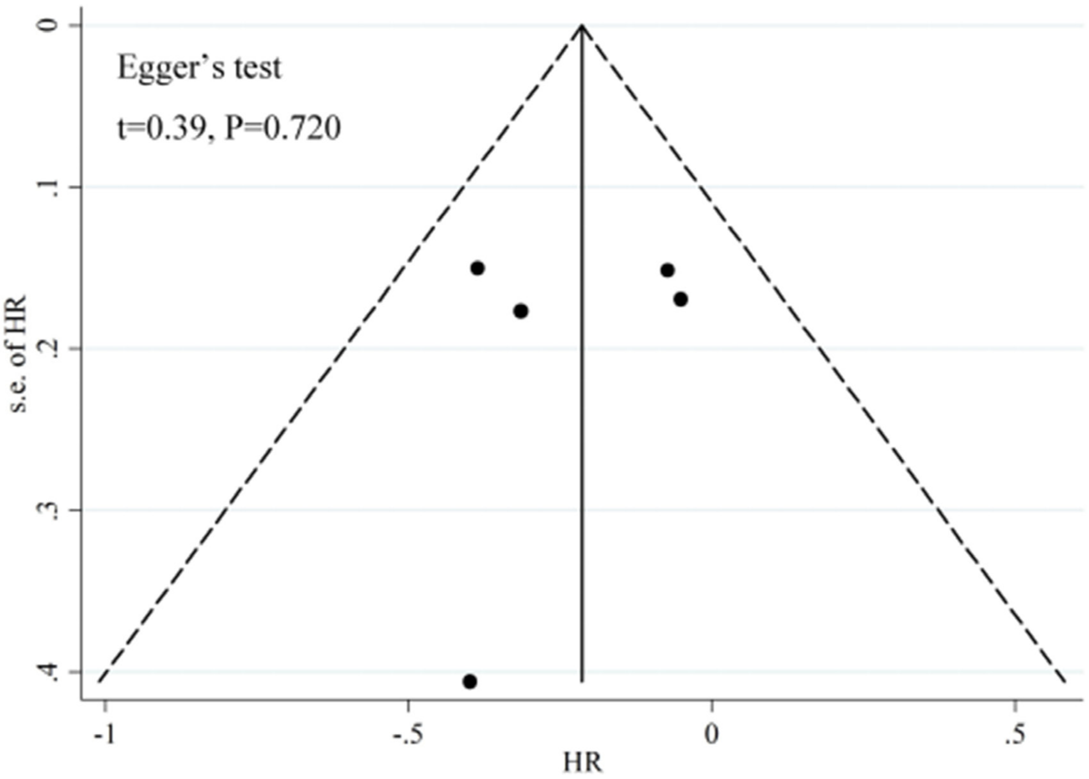

Supplementary Figure 2: Funnel plot of publication bias in OS for the included studies.
